# STING deficiency alleviates scar formation after glaucoma filtration surgery by suppressing p38 MAPK-induced inflammation in mice

**DOI:** 10.1186/s40662-026-00475-3

**Published:** 2026-02-02

**Authors:** Huifang Ye, Xinlei Lu, Hongjin Chen, Xi Yang, Han Xu, Lei Huang, Qinxiang Zheng, Rongrong Le, Yuanbo Liang

**Affiliations:** 1 State Key Laboratory of Ophthalmology, Optometry and Visual Science, Eye Hospital, Wenzhou Medical University, Wenzhou, 325027 China; 2 National Clinical Research Center for Ocular Diseases, Eye Hospital, Wenzhou Medical University, Wenzhou, Zhejiang 325027 China; 3 Center for Tissue Engineering and Stem Cell Research, Translation Medicine Research Center, Guizhou Biomanufacturing Laboratory, Guizhou Medical University, Guiyang, 561113 China; 4 Department of Ophthalmology, Zhongshan Hospital, Fudan University, Shanghai, 200032 China; 5 Department of Ophthalmology, Sir Run Run Shaw Hospital, Zhejiang University School of Medicine, Hangzhou, 310000 China

**Keywords:** STING, Glaucoma, p38, H151, Scar, Fibrosis, Tenon’s fibroblasts, Inflammation

## Abstract

**Background:**

Glaucoma filtration surgery (GFS) often fails because of excessive scar formation driven by inflammation and fibroblast activation. Although the stimulator of interferon genes (STING) pathway is involved in inflammatory responses, its role in post-surgical fibrosis remains unclear.

**Methods:**

A mouse GFS model was established in wild-type (WT) and STING-knockout (STING-KO) animals. A parallel cohort of WT mice received a single intraoperative subconjunctival injection of the STING inhibitor H151. Bleb survival, intraocular pressure, histopathology, collagen deposition, and inflammatory/fibrotic markers were evaluated for 28 days. RNA sequencing, Western blotting, and ELISA were employed to profile the p38/MAPK axis. Primary human Tenon’s capsule fibroblasts were treated with angiotensin II in the presence or absence of STING silencing or H151 to corroborate mechanisms in vitro.

**Results:**

STING expression was markedly up-regulated in fibroblasts within human and mice post-GFS tissues. STING-KO mice exhibited prolonged bleb survival together with reduced collagen deposition and fibroblast activation. RNA-sequencing revealed that STING deletion significantly altered the p38 mitogen-activated protein kinase (MAPK) pathway. Mechanistically, STING deficiency suppressed p38 MAPK phosphorylation, leading to decreased levels of the pro-inflammatory cytokines interleukin-6 (IL-6), tumor necrosis factor-α, IL-18, and IL-1β, as well as the fibrogenic factors α-SMA, collagen I, fibronectin, connective tissue growth factor, and collagen type III alpha 1 at the surgical sites. Consistently, the selective STING inhibitor H151 recapitulated these effects by suppressing p38 MAPK signaling and markedly reducing fibrotic scarring.

**Conclusions:**

STING deficiency alleviates scar formation after GFS by suppressing p38 MAPK pathway. Targeting STING/p38 axis may improve surgical outcomes by modulating the balance between inflammation and tissue repair.

**Supplementary Information:**

The online version contains supplementary material available at 10.1186/s40662-026-00475-3.

## Background

Glaucoma is a progressive optic neuropathy characterized by the irreversible degeneration of retinal ganglion cells and corresponding visual field loss, being one of the leading causes of irreversible blindness worldwide [1]. Elevated intraocular pressure (IOP), a major pathological hallmark of glaucoma, induces mechanical compression and ischemic injury to the optic nerve head [2]. Glaucoma filtration surgery (GFS) remains the primary intervention for reducing IOP and preserving vision in advanced cases refractory to medical therapy [3]. However, approximately 50% of patients experience surgical failure within five years, mainly due to excessive subconjunctival scarring at the surgical site [4, 5]. Although adjunctive antiproliferative agents such as mitomycin C and 5-fluorouracil can transiently inhibit fibroblast proliferation [6–8], their nonspecific cytotoxicity damages surrounding ocular tissues, resulting in conjunctival or scleral thinning, necrosis and a range of postoperative complications including hypotony, shallow anterior chamber, choroidal detachment, hypotony maculopathy and ocular surface damage (including corneal and conjunctival epithelial defects, keratoconjunctivitis, endophthalmitis and conjunctival wound leakage) [9–11]. Even with these agents, the long-term surgical success rate remains only 40%–67% [12–14]. Consequently, reducing bleb scarring is essential for improving surgical outcomes, and a deeper understanding of the underlying mechanisms and therapeutic targets is critical for developing more effective antifibrotic strategies in GFS.

Immune-mediated fibrotic pathways have recently emerged as an important research hotspot. Stimulator of interferon genes (STING), a key regulator of innate immune activation, has been increasingly implicated in the pathogenesis of various chronic diseases [15, 16]. As a cytosolic DNA sensor, STING plays a central role in detecting abnormal intracellular DNA and initiating downstream inflammatory signaling [17]. Recent studies have gradually revealed that STING-related pathways contribute to fibrosis mediated by chronic inflammation in multiple organs and tissues. Han et al. reported that angiotensin II (Ang II) induces the abnormal activation of the gasdermin D-STING (GSDMD-STING) axis, leading to cardiomyocyte injury, inflammation, and fibrosis [16]. In addition, Zhang et al. reported that the activation of the cyclic GMP-AMP synthase-STING (cGAS-STING) pathway in retinal ganglion cells is associated with DNA damage, inflammation, and mitochondrial dysfunction [18]. Similarly, Tanaka et al. showed that the selective STING inhibitor H151 reduces choroidal neovascularization in a murine model of age-related macular degeneration, suggesting that the suppression of cGAS-STING attenuates ocular inflammation and vascular leakage [19].

Although the role of STING in chronic fibrotic diseases has been partially elucidated, its downstream effectors have not been investigated in the context of GFS, and the STING-effector axis has not been pharmacologically targeted to modulate postoperative scarring. Its role in acute wound healing after GFS remains unclear. The immediate postoperative microenvironment, characterized by damage-associated molecular patterns (DAMPs) released from surgical trauma, leukocyte infiltration, and hypoxia-reperfusion injury, creates conditions in which STING signaling may critically influence the early transition from inflammation to fibrosis. Therefore, this study aimed to elucidate the role of STING in scar formation associated with inflammatory signaling after GFS using a mouse model, including STING-knockout (STING-KO) mice, to identify potential therapeutic targets for modulating postoperative immune response in GFS.

## Methods

### Reagents and chemicals

H151 (HY-112693) and SB203580 (HY-10256) were purchased from MedChemExpress (Monmouth Junction, NJ, USA). Ang II was purchased from Sigma-Aldrich (A9252, St. Louis, MO, USA). The following antibodies were used for Western blotting: anti-phospho-p38 (28796-1-AP, ProteinTech, China), anti-p38 (14064-1-AP, ProteinTech, China), anti-α-smooth muscle actin (α-SMA) (14395-1-AP, ProteinTech, China), anti-collagen I (14695-1-AP, ProteinTech, China), and anti-STING (13647, Cell Signaling Technology, USA). The following antibodies were used for immunofluorescence and immunohistochemistry: anti-STING (13647, Cell Signaling Technology, USA), anti-α-SMA (14395-1-AP, ProteinTech, China), anti-phospho-tank-binding kinase 1 (TBK1) (5483, Cell Signaling Technology, USA), anti-phospho-interferon regulatory factor 3 (IRF3) (29047, Cell Signaling Technology, USA), anti-phospho-p65 (3033, Cell Signaling Technology, USA), and anti-F4/80 (R013726, Epizyme, China).

### Mouse model

All experimental procedures were approved by the Ethics Committee of Wenzhou Medical University (approval number: WYDW-2021-0576), and in accordance with the Statement for the use of animals from the Association for Research in Vision and Ophthalmology.

C57BL/6J wild-type (WT) mice were provided by the Animal Center of Wenzhou Medical University, while the STING KO (*Sting*^*−/−*^) mice were purchased from the Jackson Laboratory (025805, MPYS). GFS was performed in WT and *Sting*^*−/−*^ male mice (7–8 weeks old) according to a previous study [20]. All mice were randomly divided into groups; details are described in the Supplementary Materials. Mice were anesthetized with pentobarbital sodium (50 mg/kg), and a conjunctival incision was made on one eye to create a small filtration space in the subconjunctival region. Subsequently, a fistula was created by inserting a 30-gauge needle through the sclera into the anterior chamber, allowing aqueous humor to drain into the subconjunctival region. The conjunctiva was subsequently closed over the fistula using a 10/0 nylon suture. With regards to WT mice, a subconjunctival injection of H151 (1 mM) or dimethyl sulfoxide was administered according to group assignment. Levofloxacin eye drops (0.5%) were applied postoperatively to prevent infection. Imaging of the operated eyes was performed at baseline and on postoperative days 7, 14, and 28, and filtering blebs were scored using the Indiana Bleb Appearance Grading Scale (IBAGS). IOP was measured at baseline, immediately after surgery, and on days 7 and 28. Eyes were harvested on day 28 after surgery.

### Cell culture

Human Tenon’s capsule fibroblasts (HTFs) were cultured using an explant tissue culture method as previously described [21]. Tenon’s capsule tissue was obtained during strabismus surgeries of six patients (three males and three females), with no history of ocular surgery, uveitis, trauma, or chronic tropical medication use. All procedures involving human subjects were approved by the Ethics Committee of the Eye Hospital, Wenzhou Medical University (approval number: 2021-246-K-214-02) and were performed according to the Declaration of Helsinki. HTFs were cultured in DMEM/F12 supplemented with 10% fetal bovine serum and 1% penicillin–streptomycin and incubated at 37 °C under 5% CO_2_ conditions. HTFs at passages 5–6 were used for all experiments.

### Gene silencing and overexpression

HTFs were seeded in a 6-well plate at a density of 1 × 10^5^ cells/well and cultured until they reached 75%–80% confluence. For gene silencing, 2.5 µL endoribonuclease-prepared small interfering RNA (esiRNA) (HU33481, Sigma-Aldrich) specifically targeting STING (experimental wells) or 2.5 µL negative control (NC) esiRNA (control wells) were added to 200 µL jetPRIME buffer (101000046, Polyplus) and vortexed thoroughly. Next, jetPRIME reagent (4 µL) was added to each well and incubated for 15 min at room temperature. Subsequently, transfection complex (200 µL) was added into the corresponding wells of the 6-well plate, which was gently shaken to ensure uniform distribution. Cells were harvested for subsequent assays after 24 h transfection. For gene overexpression, STING expression plasmids under the control of a strong promoter were generated using standard molecular cloning techniques. Cells were transfected with these plasmids according to the manufacturer’s instructions and were cultured for 24 h post-transfection before being used for further experiments.

### Real-time reverse transcription-quantitative polymerase chain reaction (RT-qPCR)

Total RNA was extracted from cells or eye tissues using the Animal RNA Isolation Kit with Spin Column (R0024, Beyotime, China), following the manufacturer’s instructions. cDNA was synthesized from 1 µg total RNA using the PrimeScript RT reagent kit (RR036, Takara, Otsu, Japan). RT-qPCR was performed using the SYBR Green Master Mix (K1070, APExBIO, USA) on an Applied Biosystems QuantStudio 6 Flex Real-Time PCR Detection System (ThermoFisher Scientific, MA, USA). Cycling conditions included initial denaturation at 95 °C for 10 min followed by 40 cycles at 95 °C for 15 s and 60 °C for 1 min. Relative gene expression was calculated using the 2^−ΔΔCt^ method with glyceraldehyde 3-phosphate dehydrogenase (GAPDH) as the internal control. Primer sequences used in this study can be found in Additional file 1: Table S1.

### Western blotting

HTFs were lysed in radioimmunoprecipitation (RIPA) buffer and proteins were extracted, quantified, separated using sodium dodecyl sulfate–polyacrylamide gel electrophoresis (SDS-PAGE), and transferred to a polyvinylidene difluoride (PVDF) membrane. Non-specific proteins were blocked with 5% non-fat skim milk in Tris-buffered saline with tween 20 (TBST) buffer, and the membrane was incubated with primary antibodies at 4 °C overnight. Subsequently, the membrane was incubated with horseradish peroxidase (HRP)-conjugated secondary antibody for 1 h at 37 °C. Finally, proteins were detected using an enhanced chemiluminescence (ECL) reagent. Images were taken, and the number of proteins was analyzed and normalized to the respective internal control using ImageJ software (National Institutes of Health, Bethesda, MD, USA).

### Wound healing assay

HTFs were seeded in 6-well plates until they reached 90% confluence. A sterile 100-µL pipette tip was used to create a straight-line scratch in the center of the cell monolayer. Cells were washed three times with sterile Dulbecco’s phosphate-buffered saline so that the scratch was clearly visible. The culture medium was then replaced with a fresh serum-free medium with or without H151 for 2 h. Subsequently, Ang II was added to all groups except the control. Cells were observed and photographed under a microscope (Axio Observer 3, Zeiss, Germany) at different time points including baseline, 12 h, and 24 h. The migrated cells were quantified using ImageJ software (National Institutes of Health, Bethesda, MD, USA).

### RNA sequencing (RNA-seq)

Mice were anesthetized, the Tenon’s tissue under the conjunctiva was removed, and the blood on the surface of the tissue was quickly washed with pre-cooled physiological saline. Subsequently, the processed tissue was stored in RNA later (R0901, Sigma-Aldrich) according to the manufacturer’s instructions. Transcriptomic data processing was performed by Biozeron biotechnology (Shanghai, China) using the CFViSA platform [22], an integrated system that provides standardized analytical workflows, and with approximately 80 specialized tools for sequence processing, visualization, and statistical analysis. Gene expression was quantified using the fragments per kilobase of exon per million mapped reads (FRKM) method [23], enabling cross-sample comparisons. Differential gene expression profiling among experimental groups was performed using the edgeR package [24] in R/Bioconductor, with significance defined as |log_2_ fold change|> 2 and false discovery rate-adjusted *P* < 0.05. Functional annotation of differentially expressed genes was performed using two complementary approaches: Gene Ontology enrichment analysis using the Goatools Python module [25], and pathway enrichment analysis using the KOBAS platform [26]. Additionally, comprehensive gene set evaluation was performed by Gene Set Enrichment Analysis using the molecular signatures database (https://software.broadinstitute.org/gsea/msigdb).

### Histological staining

Eye tissues from mice and human fascia tissue were fixed in 10% formaldehyde for 24 h. Hematoxylin and eosin (H&E) staining was performed on paraffin-embedded eye sections to observe their histological features. Masson’s trichrome staining (G1340, Solarbio, China) and Sirius red staining (G1078, Servicebio, China) were performed according to the manufacturer’s instructions to observe the collagen condition. Images were acquired using a light microscope (Ni-U, Nikon, Japan).

### Immunofluorescence staining

Eye tissue samples from mice and HTFs were fixed in 4% paraformaldehyde at 4 °C for 30 min, followed by dehydration overnight. Subsequently, the eye tissue/HTF samples were embedded in optimal cutting temperature compound, cut into 12 µm-thick sections using a microtome, and placed on glass slides. The eye tissue/HTF sections were permeabilized using 0.1% Triton X-100 for 5 min and blocked using 10% goat serum at 37 °C for 1 h. Sections were then incubated with primary antibodies overnight at 4 °C. Subsequently, they were incubated with secondary antibodies for 1 h at 37 °C and stained with 4′,6-diamidino-2-phenylindole (DAPI). Images were captured using a microscope (Axio Observer 3, Zeiss, Germany).

### Immunohistochemical staining

Immunohistochemical staining was performed on paraffin-embedded eye tissue sections from mice and human fascia tissue sections. Human fascia samples were collected from Tenon’s capsule tissue of six patients who underwent either strabismus surgery (n = 3, Sham group) or enucleation/secondary glaucoma surgery (n = 3, surgery group) due to scarring following previous GFS. Sections were deparaffinized in xylene and rehydrated through a graded ethanol series. Antigen retrieval was performed in citrate buffer (pH 6.0) using heat-induced epitope retrieval. Sections were blocked with 10% goat serum for 1 h at room temperature, followed by overnight incubation at 4 °C with primary antibodies. Sections were washed with phosphate buffered saline (PBS), incubated with HRP-conjugated secondary antibodies for 1 h at room temperature, and the staining was visualized using 3,3′-diaminobenzidine (DAB). Nuclei were counterstained with hematoxylin, and sections were mounted with neutral gum and imaged using a microscope (Ni-U, Nikon, Japan).

### Enzyme-linked immunosorbent assay (ELISA)

Human fascia tissues and HTFs were collected and analyzed using commercial ELISA kits for human Ang II (E-EL-H0326, Elabscience, China), human interleukin (IL)-6 (HJ064, Epizyme, China), and human tumor necrosis factor-α (TNF-α) (HJ110, Epizyme, China), following the manufacturer’s instructions.

### Statistical analysis

Statistical analysis was performed using GraphPad Prism 10.4.2 software (GraphPad Inc., Boston, Massachusetts, USA). Data normality was assessed using the Shapiro–Wilk’s test, while homoscedasticity was evaluated using the F test or Brown-Forsythe test. For the comparison between two groups, normally distributed data were analyzed using the unpaired-Student’s *t*-test, whereas Welch’s *t*-test was used in the presence of heteroscedasticity. Non-normally distributed data were analyzed using the Mann–Whitney U test. For the comparison of multiple groups, one-way analysis of variance (ANOVA) was used for normally distributed data, while two-way ANOVA was used when more than one independent variable was considered. When ANOVA indicated a significant difference, Bonferroni’s post hoc test was performed for pairwise comparisons. Welch’s ANOVA followed by Dunnett’s T3 post hoc test was used for normally distributed data with unequal variances. For non-normally distributed data involving more than two groups, the Kruskal–Wallis test and Wilcoxon signed-ranks test with Bonferroni correction was used. Bleb survival was analyzed using the Kaplan–Meier survival curves and compared using the log-rank test. Results were expressed as mean ± SD or median (interquartile range). A value of *P* < 0.05 was considered statistically significant.

## Results

### STING overexpression in HTFs is associated with postoperative scarring after GFS

**Fig. 1:**
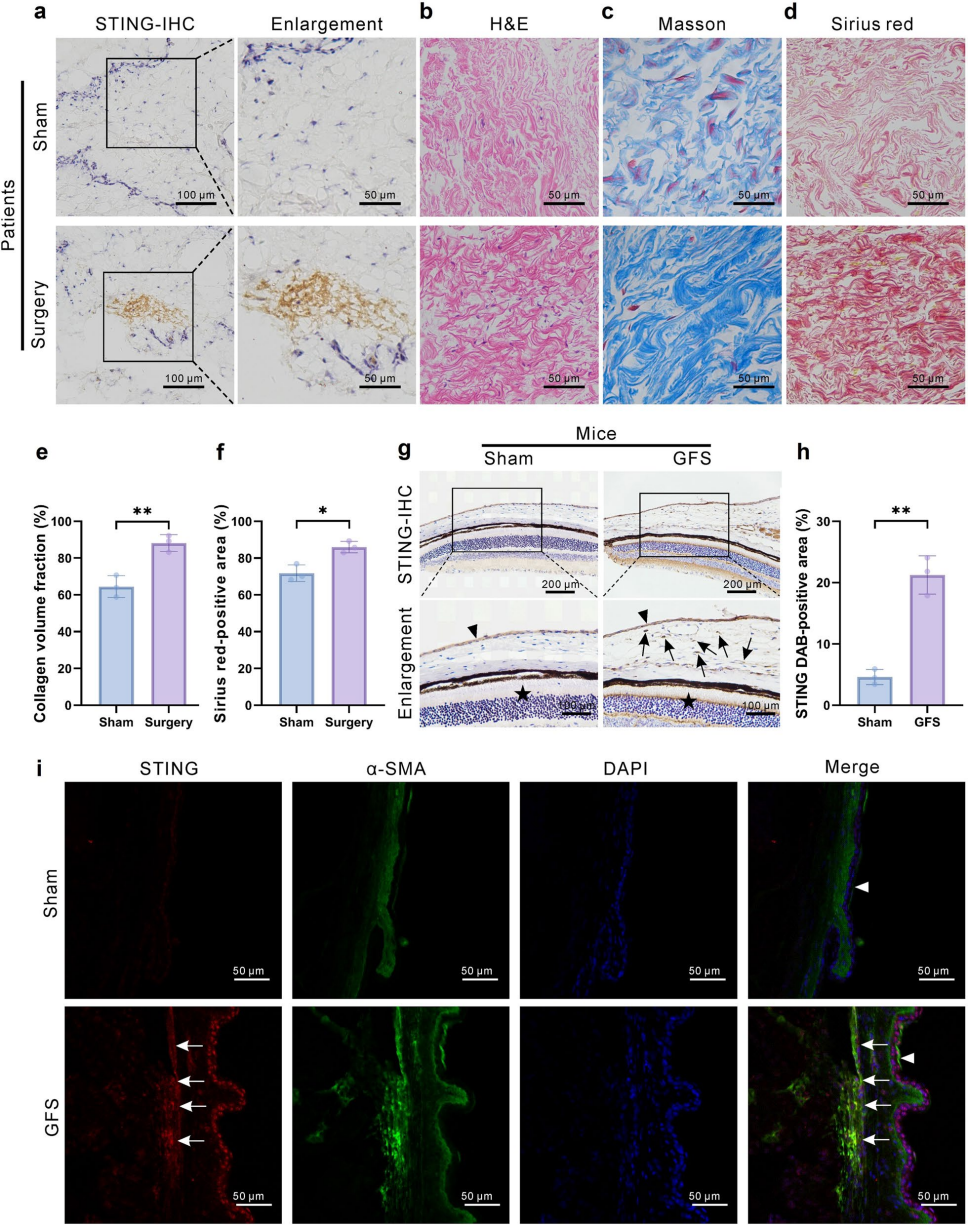
Immunohistochemical analysis of STING in the sub-Tenon’s capsule tissue of the conjunctiva. **a** Representative images of STING staining in the sub-Tenon’s capsule tissue from Sham (untreated) and glaucoma filtration surgery (GFS) groups (n = 3 eyes in each group). **b** Hematoxylin and eosin (H&E) staining of tissue sections from the Sham and GFS groups (n = 3 eyes in each group). **c** Masson staining of tissue sections. **d** Sirius red staining of tissue sections; **e** Analysis of Masson staining (n = 3 eyes in each group). **f** Analysis of Sirius red staining (n = 3 eyes in each group). **g** Histological sections of the Sham and GFS groups of mice showing the staining of the tissue. The triangles indicate the conjunctiva, while the stars indicate the retina. Arrows indicate the positive area. **h** Quantitative analysis of STING DAB-positive area (n = 3 eyes in each group). **i** Double immunofluorescence staining of STING (red), α-SMA (green), and DAPI (blue) in the Sham and GFS groups (n = 3 eyes in each group). The triangles indicate the conjunctiva. Arrows indicate the co-localization of STING and α-SMA in the GFS group. Results are expressed as mean ± SD. Statistical analysis was performed using unpaired Student’s *t*-test in (**e**, **f**, **h**). *P < 0.05, **P < 0.01. STING, stimulator of interferon genes; Sham, sham-operated; α-SMA, α-smooth muscle actin; DAPI, 4′,6-diamidino-2-phenylindole

STING expression was examined in conjunctival Tenon’s capsule tissues from individuals without prior ocular surgery and from individuals with filtering bleb scarring after GFS to elucidate the role of STING in postoperative scarring after GFS. STING expression was significantly increased in conjunctival tissues with filtering bleb scarring after surgery (Fig. 1a). H&E staining showed that the conjunctival tissues became denser and more disorganized after surgery (Fig. 1b). Masson and Sirius red stainings further revealed a significant increase in collagen deposition in tissues with filtering bleb scarring (Fig. 1c–f). A mouse model of glaucoma with postoperative scarring was then established using a 30-gauge needle as previously described [20]. Immunohistochemical analysis of conjunctival Tenon’s capsule tissues collected 4 weeks after surgery revealed high STING expression in the surgical group (Fig. 1g, h). Previous studies showed that fibroblasts contribute to scarring after GFS, and STING is also expressed in these cells. Immunofluorescence staining was performed using α-SMA as a marker for HTFs to determine the cellular source of STING in conjunctival tissues. STING immunoreactivity was colocalized in α-SMA-positive regions in mouse HTFs (Fig. 1i), suggesting that STING overexpression during postoperative scarring was largely localized into fibroblasts. Collectively, these findings suggested that STING acted as a potential regulator of postoperative scarring in glaucoma.

### STING deficiency protects against postoperative scarring after GFS

**Fig. 2:**
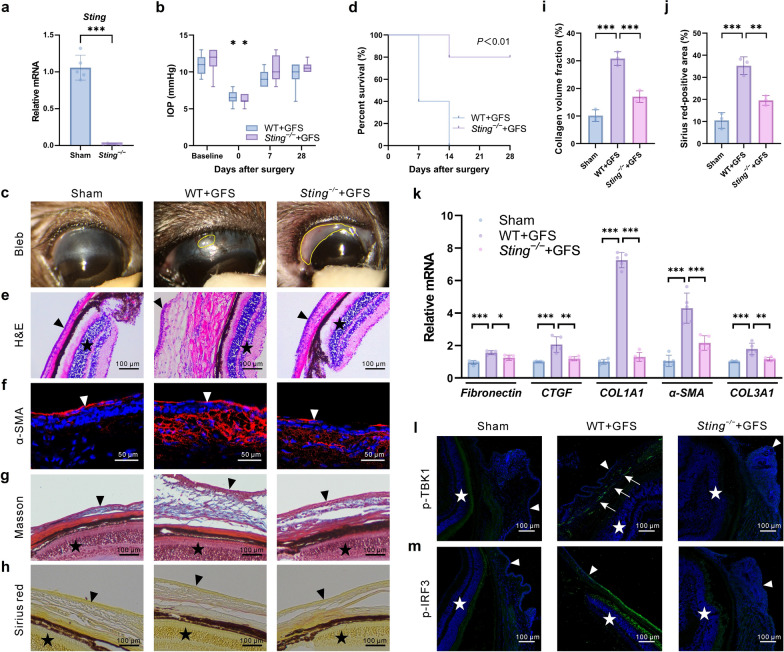
In vivo effects of *Sting* gene knockout on inflammation and fibrosis after glaucoma filtration surgery (GFS). **a** Relative *Sting* mRNA expression in the Sham and *Sting*^*−/−*^ groups (n = 5 eyes in each group). **b** Intraocular pressure (IOP) changes in the WT + GFS and *Sting*^*−/−*^ + GFS groups over 28 days post-surgery (n = 10 eyes in each group). **c** Representative images of bleb morphology in the Sham, WT + GFS, and *Sting*^*−/−*^ + GFS groups. Yellow circles indicate the blebs. **d** Bleb survival analysis in the WT + GFS and *Sting*^*−/−*^ + GFS groups using Kaplan–Meier survival curves (n = 5 eyes in each group). **e** Hematoxylin and eosin (H&E) staining of tissue sections from the Sham, WT + GFS, and *Sting*^*−/−*^ + GFS groups (n = 3 eyes in each group). The triangles indicate the conjunctiva, the stars indicate the retina. **f** Immunofluorescence staining of α-SMA in the Sham, WT + GFS, and *Sting*^*−/−*^ + GFS groups (n = 3 eyes in each group). The triangles indicate the conjunctiva. **g** Masson staining of tissue sections from the Sham, WT + GFS, and *Sting*^*−/−*^ + GFS groups. The triangles indicate the conjunctiva, the stars indicate the retina. **h** Sirius ed staining of tissue sections from the Sham, WT + GFS, and *Sting*^*−/−*^ + GFS groups. The triangles indicate the conjunctiva, the stars indicate the retina. **i** Analysis of Masson staining (n = 3 eyes in each group). **j** Analysis of Sirius red staining (n = 3 eyes in each group). **k** Relative mRNA expression of fibronectin, CTGF, COL1A1, α-SMA, and COL3A1 in the Sham, WT + GFS, and *Sting*^*−/−*^ + GFS groups (n = 5 eyes in each group). **l**, **m** Immunofluorescence staining of p-TBK1 and p-IRF3 in the Sham, WT + GFS, and *Sting*^*−/−*^ + GFS groups (n = 3 eyes in each group). The triangles indicate the conjunctiva, the stars indicate the retina. Arrows indicate the p-TBK1 expression area. Results were expressed as mean ± SD except (**b**) that was expressed as median (lower to upper quartile values). Statistical analysis was performed using unpaired *t*-test with Welch’s correction in (**a**), Wilcoxon signed-ranks test with Bonferroni correction in (**b**), log-rank test in (**d**), and one-way ANOVA followed by Bonferroni’s post-hoc test in (**i**, **j**, **k**). *P < 0.05, **P < 0.01, ***P < 0.001. Sham, sham-operated; WT, wild-type; α-SMA, α-smooth muscle actin; CTGF, connective tissue growth factor; COL1A1, collagen type I alpha 1; COL3A1, collagen type III alpha 1; p-TBK1, phospho-tank-binding kinase 1; p-IRF3, phospho-interferon regulatory factor 3

*Sting*^*−/−*^ mice were generated, and successful *Sting* knockout in conjunctival tissues was confirmed by RT-qPCR (Fig. 2a). A mouse model of GFS was then established to evaluate the effect of STING deficiency on Tenon’s capsule scarring. IOP did not differ significantly between WT and *Sting*^*−/−*^ mice before surgery, but it significantly decreased immediately after surgery in both groups, indicating the successful modeling (Fig. 2b). Note that IOP is the pressure exerted by the content of the eye on the eyeball wall, an essential examination for the diagnosis and treatment of glaucoma. *Sting* knockout did not influence IOP changes in mice at any postoperative time point. During the early postoperative period, mice in all groups exhibited prominent filtering blebs, which gradually flattened by 4 weeks, indicating progressive Tenon’s capsule scarring. However, bleb survival was significantly prolonged in *Sting*^*−/−*^ mice compared with WT mice, indicating a slower scaring process (Fig. 2c, d). Bleb characteristics in both groups were evaluated using the IBAGS [27], with detailed scores presented in the supplementary material. H&E staining showed a significant thickening of the conjunctival Tenon’s capsule in WT mice after surgery (Fig. 2e). Furthermore, α-SMA fluorescence staining showed a significant increase in fibrosis in WT mice postoperatively (Fig. 2e), whereas this response was attenuated in *Sting*^*−/−*^ mice. Masson and Sirius red stainings further confirmed the significantly increased collagen deposition in WT mice after surgery, a change that was significantly reduced in *Sting*^*−/−*^ mice (Fig. 2g–j). Mouse conjunctival Tenon’s capsule tissue was collected to detect fibrosis, revealing that *Sting*^*−/−*^ inhibited the mRNA expression of several fibrosis factors induced by surgery, such as connective tissue growth factor (CTGF), collagen type Ⅰ alpha 1 (COL1A1), collagen type III alpha 1 (COL3A1), α-SMA, and fibronectin (Fig. 2k). TBK1 and IRF3 promote glaucoma progression. Thus, fluorescence staining was performed to detect the phosphorylation of TBK1 and IRF3, which are known classic downstream targets of STING. The phosphorylation of TBK1 and IRF3 significantly increased after GFS, whereas *Sting* knockout reduced their phosphorylation (Fig. 2l, m). Taken together, these findings indicated that *Sting* deficiency protected against postoperative scarring after GFS.

### STING knockout inhibits inflammation after GFS

**Fig. 3:**
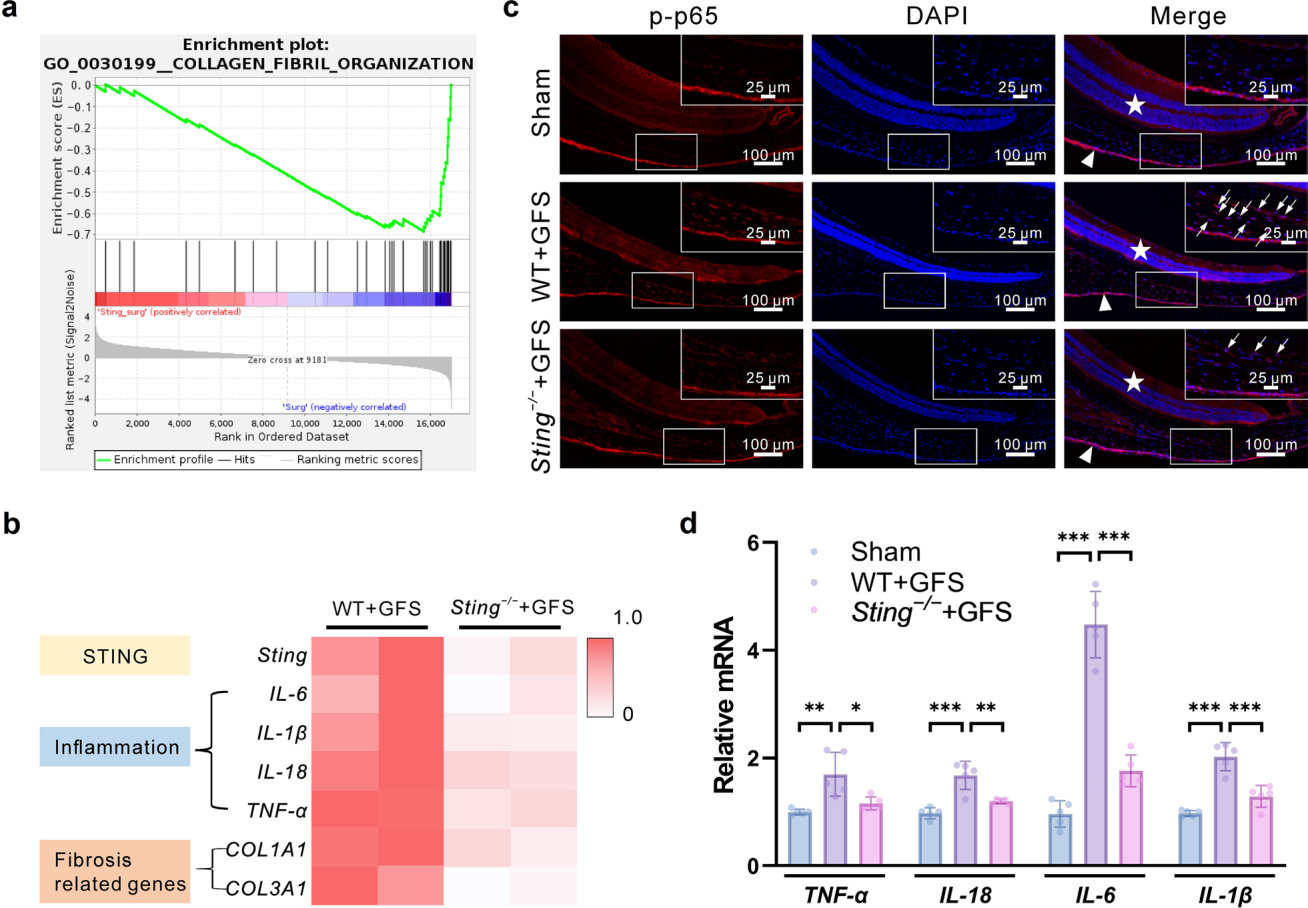
*Sting* knockdown inhibited GFS-induced inflammation. **a** Gene set enrichment analysis of the enrichment of differentially expressed genes between *Sting*^*−/−*^ and matched normal in collagen fibril organization. **b** Heatmaps derived from RNA sequencing data indicated that *Sting*^*−/−*^ inhibited the expression of key genes involved in inflammation and fibrosis. **c** Immunofluorescence staining of p-p65 in Tenon’s capsule tissues (n = 3 eyes in each group). The triangles indicate the conjunctiva, the stars indicate the retina. Arrows indicate the p-p65 expression area. **d** Relative mRNA expression of TNF-α, IL-18, IL-6, and IL-1β in the Sham, WT + GFS, and *Sting*^*−/−*^ + GFS groups (n = 5 eyes in each group). Results were expressed as mean ± SD. Statistical analysis was performed using one-way ANOVA followed by Bonferroni’s post-hoc test in (**d**). *P < 0.05, **P < 0.01, ***P < 0.001. GFS, glaucoma filtration surgery; p-p65, phospho-p65; TNF-α, tumor necrosis factor-α; IL, interleukin; Sham, sham-operated; WT, wild-type; DAPI, 4′,6-diamidino-2-phenylindole

RNA sequencing was performed on Tenon’s capsule tissues isolated from WT and *Sting*^*−/−*^ mice after GFS. *Sting*^*−/−*^ inhibited key inflammatory molecules and fibrosis-related genes (Fig. 3a, b). The principal component analysis (PCA) of RNA sequencing and the correlation heatmap are shown in Additional file 1: Fig. S1. The results showed a significant increase in the phosphorylation of p65 in the conjunctival Tenon’s capsule tissues of postoperative WT mice (Fig. 3c), as well as the expression of *IL-6*, *IL-18*, *IL-1β*, and *TNF-α* (Fig. 3d). In contrast, STING deficiency suppressed p65 phosphorylation (Fig. 3c) and *IL-6*, *IL-18*, *IL-1β*, and *TNF-α* mRNA expression in conjunctival Tenon’s capsule tissues after surgery (Fig. 3d).

### Glaucoma-mediated Ang II induces STING activation

**Fig. 4:**
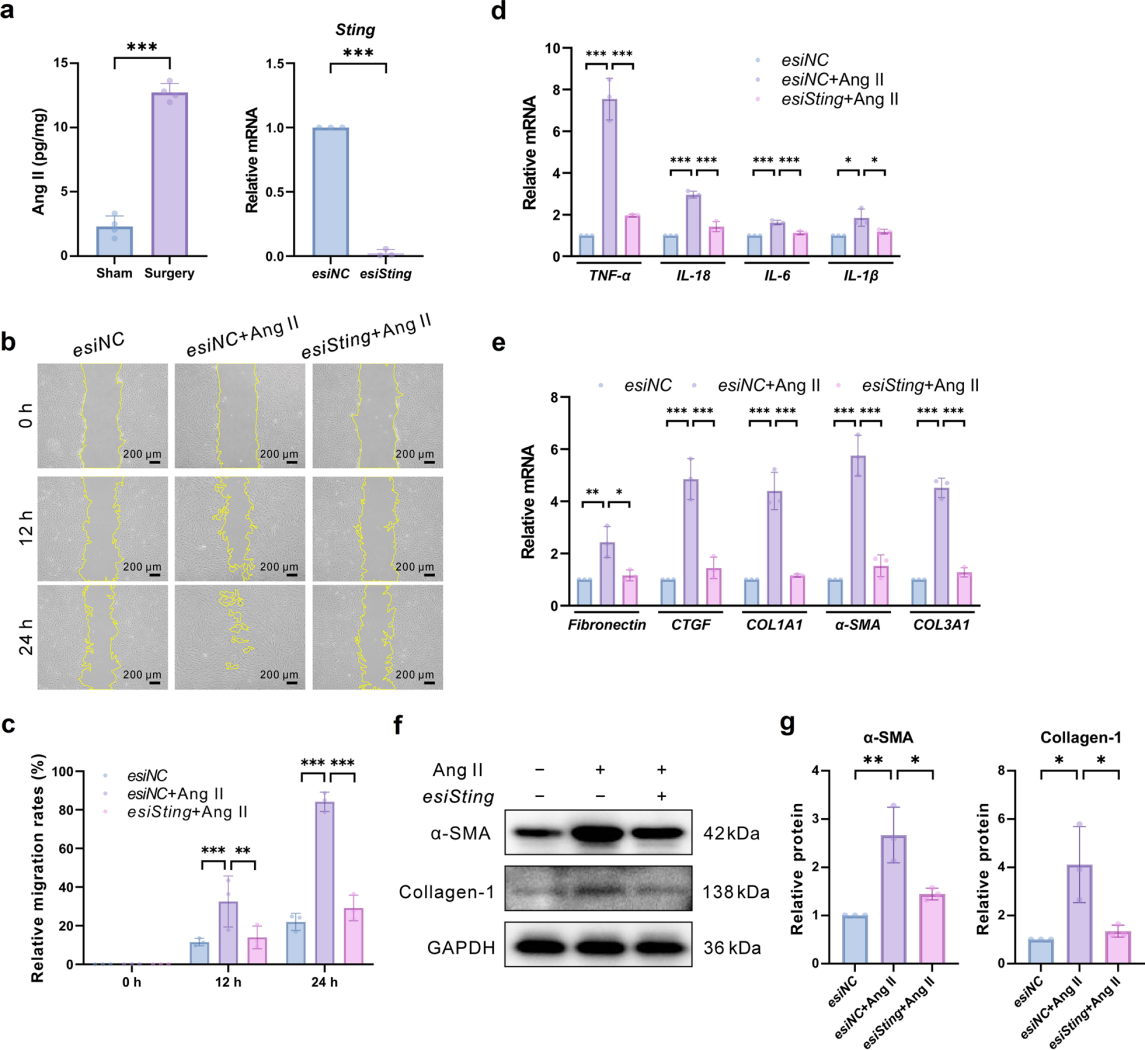
In vitro effects of *Sting* gene knockout on Ang II-induced inflammation and fibrosis of HTFs. **a** Concentration of Ang II in the sub-Tenon’s capsule tissue from Sham (untreated) and surgery (glaucoma filtration surgery) patients determined by ELISA (n = 4 eyes in each group). *Sting* mRNA expression in HTFs after *Sting* silencing (n = 3 eyes in each group). **b**, **c** Wound healing assay: cells were incubated with or without *esiSting* 24 h before Ang II (1 μM) treatment for 24 h. Subsequently, wound scratches were imaged and quantified (n = 3 eyes in each group). **b** Representative images of the different treatment groups at different time points after the scratch. **c** Relative migration rates in each group. **d** Relative mRNA expression of TNF-α, IL-18, IL-6, and IL-1β in *esiNC*, *esiNC* + Ang II, and *esiSting* + Ang II groups (n = 3 eyes in each group). **e** Relative mRNA expression of fibronectin, CTGF, COL1A1, α-SMA, and COL3A1 in *esiNC*, *esiNC* + Ang II, and *esiSting* + Ang II groups (n = 3 eyes in each group). **f**, **g** Representative immunoblotting images: *Sting* silencing significantly inhibited Ang II-induced α-SMA and collagen I protein expression by western blot (n = 3 eyes in each group). Results were expressed as mean ± SD. Statistical analysis was performed using unpaired Student’s *t*-test (**a** left panel), unpaired *t*-test with Welch’s correction (**a**, right panel), one-way ANOVA followed by Bonferroni’s post-hoc test (**d**, **e**, **g)**, and two-way ANOVA followed by Bonferroni’s post-hoc test (**c**). *P < 0.05, **P < 0.01, ***P < 0.001. Ang II, angiotensin II; HTFs, human Tenon’s capsule fibroblasts; TNF-α, tumor necrosis factor-α; IL, interleukin; CTGF, connective tissue growth factor; COL1A1, collagen type I alpha 1; α-SMA, α-smooth muscle actin; COL3A1, collagen type III alpha 1; GADPH, glyceraldehyde 3-phosphate dehydrogenase

Inducers for in vitro experiments were initially screened to better understand how STING participates in the downstream pathogenic mechanisms of inflammation-induced postoperative scarring in glaucoma. Previous studies showed that Ang II stimulates fibrogenesis of HTFs [28]. Furthermore, Ang II expression in the conjunctival Tenon’s capsule tissue significantly increases after GFS. Thus, the role of Ang II in postoperative scarring was assessed in conjunctival Tenon’s capsule tissues from GFS patients, revealing a significantly increased Ang II expression by ELISA (Fig. 4a). Further experiments were performed to determine whether Ang II-induced fibrosis in HTFs was regulated by STING. STING expression in HTFs was silenced using esiRNA (Fig. 4a). Wound healing assay revealed that Ang II promoted HTF migration, whereas STING silencing inhibited their migration (Fig. 4b, c). After *Sting* silencing, Ang II-induced mRNA upregulation of several inflammatory factors (TNF-α, IL-18, IL-6, and IL-1β) and fibrosis factors (fibronectin, CTGF, COL1A1, α-SMA, and COL3A1) was inhibited (Fig. 4d–l). In addition, Ang II-induced protein expression of α-SMA and collagen I was suppressed after *Sting* silencing (Fig. 4m, n). These results suggested that STING worked as a key regulatory molecule in Ang II-induced HTF migration and fibrosis.

### STING promotes the activation of the p38 mitogen-activated protein kinase (MAPK)

**Fig. 5:**
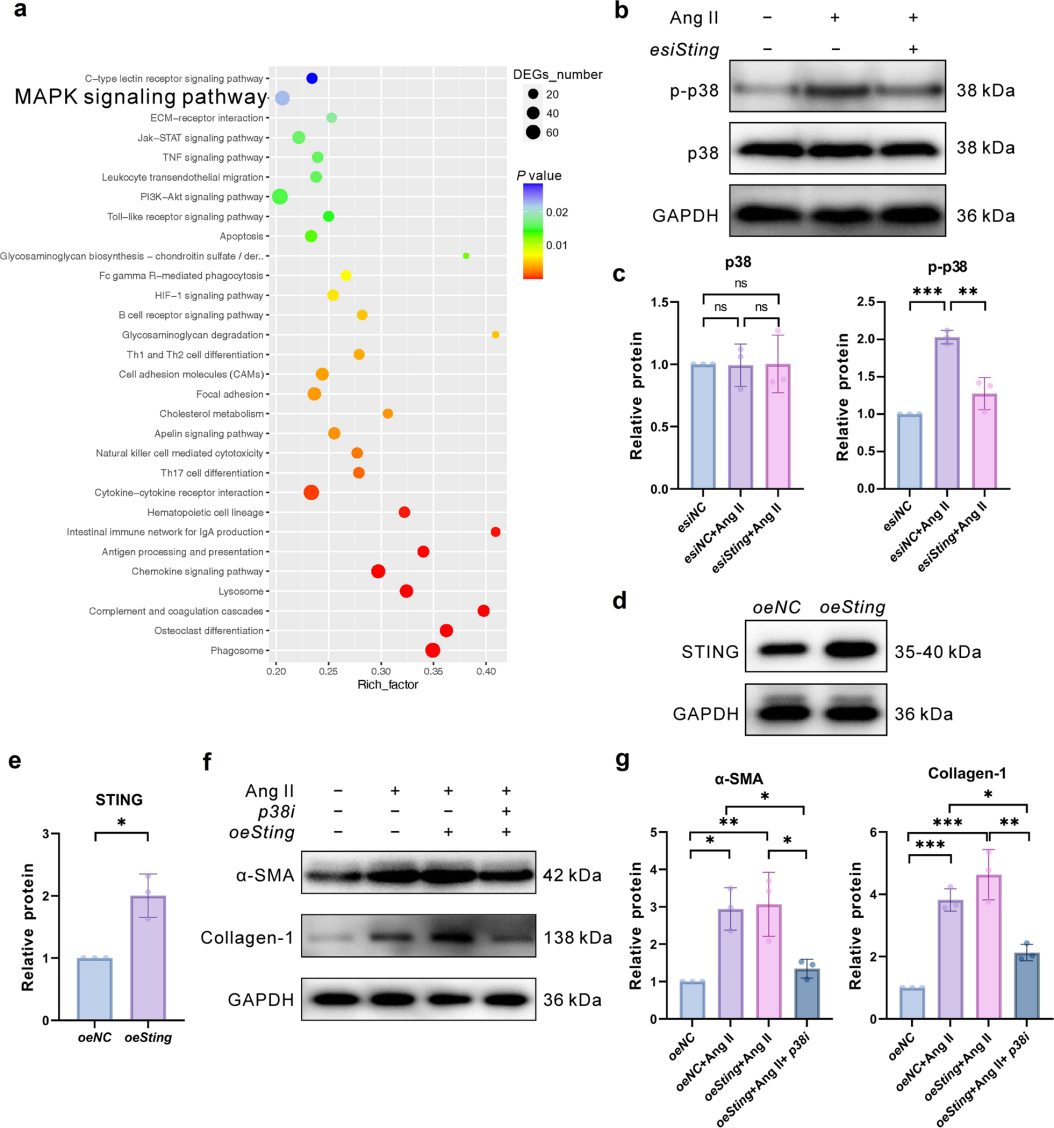
*Sting* regulated Ang II-induced p38 MAPK activation in cells. **a** KEGG analysis on the top 20 enrichment pathway identified from the differentially expressed genes between the WT + GFS and *Sting*^*−/−*^ + GFS mouse tissues. **b**, **c** Representative immunoblotting images: *esiSting* significantly inhibited Ang II-induced p-p38 protein expression by western blot (n = 3 eyes in each group). **d**, **e** Western blotting of HTFs with *Sting* overexpression (n = 3 eyes in each group). **f**, **g** Representative images of p38 inhibitor attenuating *oeSting*-induced α-SMA and collagen I expression by western blot (n = 3 eyes in each group). Results were expressed as mean ± SD. Statistical analysis was performed using unpaired *t*-test with Welch’s correction in **e** and one-way ANOVA followed by Bonferroni’s post-hoc test in **c**, **g**. ns, not significant, *P < 0.05, **P < 0.01, ***P < 0.001. Ang II, angiotensin II; p38 MAPK, p38 mitogen-activated protein kinase; KEGG, Kyoto Encyclopedia of Genes and Genomes; WT, wild-type; GFS, glaucoma filtration surgery; p-p38, phospho-p38; HTFs, human Tenon’s capsule fibroblasts; α-SMA, α-smooth muscle actin; DEG, differentially expressed genes; STING, stimulator of interferon genes; GAPDH, glyceraldehyde 3-phosphate dehydrogenase

RNA sequencing results were analyzed to investigate the role of STING in Ang II-induced fibrosis in HTFs. Kyoto Encyclopedia of Genes and Genomes (KEGG) data showed that *Sting* knockout significantly inhibited the activation of the MAPK signaling pathway (Fig. 5a). This pathway is closely associated with inflammatory responses, whereas p38 is the core pro-inflammatory molecule in the MAPK pathway. Previous studies demonstrated that STING promotes the migration and invasion of uveal melanoma cells [29]. p38 phosphorylation induced by Ang II was assessed to determine whether p38 is a key downstream effector of STING in the scar formation after GFS, revealing that Ang II promoted p-p38 expression, which was significantly inhibited by *Sting* knockout (Fig. 5b, c). The absence of p38 expression using the p38 inhibitor SB203580 resulted in the suppression of HTF migration (Additional file 1: Fig. S2a, b) and down-regulation of IL-6 and TNF-α expression (Additional file 1: Fig. S2c, d) induced by Ang II. Furthermore, STING overexpression in HTFs (Fig. 5d, e) followed by stimulation with Ang II revealed an exacerbation in the expression of the fibrosis factors α-SMA and collagen I, whereas a p38 inhibitor attenuated the expression of these fibrosis markers regardless of whether their induction resulted from Ang II stimulation or STING overexpression (Fig. 5f, g and Additional file 1: Fig. S2e, f).

### STING inhibitor H151 attenuates GFS- or Ang II-induced HTF inflammation and fibrosis in vitro and in vivo

**Fig. 6:**
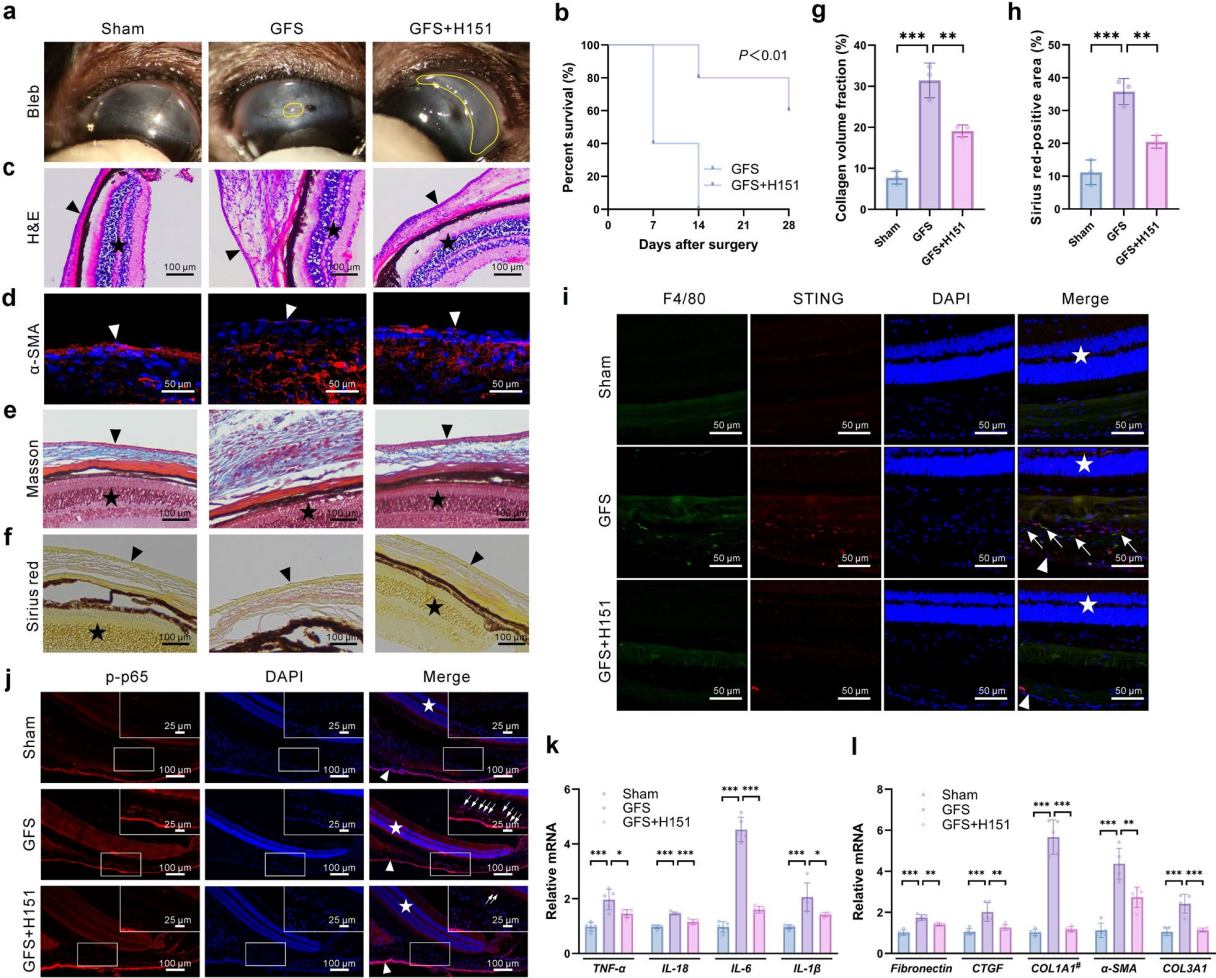
In vivo effects of the STING inhibitor H151 on proliferation after glaucoma filtration surgery (GFS). **a** Representative images from the Sham, GFS, and GFS + H151 groups. Yellow circles indicate the blebs. **b** Bleb survival analysis of the GFS and GFS + H151 groups using Kaplan–Meier survival curves (n = 5 eyes in each group). **c** Histological analysis of ocular tissues by hematoxylin and eosin (H&E) staining from the Sham, GFS, and GFS + H151 groups (n = 3 eyes in each group). The triangles indicate the conjunctiva, the stars indicate the retina. **d** Immunofluorescence staining of α-SMA to evaluate myofibroblast activation in the Sham, GFS, and GFS + H151 groups (n = 3 eyes in each group). The triangles indicate the conjunctiva. **e** Masson stained tissue sections from the Sham, GFS, and GFS + H151 groups. The triangles indicate the conjunctiva, the stars indicate the retina. **f** Sirius red staining of tissue sections from the Sham, GFS, and GFS + H151 groups. The triangles indicate the conjunctiva, the stars indicate the retina. **g** Analysis of Masson staining (n = 3 eyes in each group). **h** Analysis of Sirius red staining (n = 3 eyes in each group). **i** Double immunofluorescence staining of STING (red), F4/80 (green), and DAPI (blue) in the Sham, GFS, and GFS + H151 groups (n = 3 eyes in each group). The triangles indicate the conjunctiva, the stars indicate the retina. Arrows indicate the co-localization of STING and F4/80 in the GFS group. **j** Immunofluorescence staining of p-p65 to assess NF-κB activation in the Sham, GFS, and GFS + H151 groups (n = 3 eyes in each group). The triangles indicate the conjunctiva, the stars indicate the retina. Arrows indicate the p-p65 expression area. **k** Relative mRNA expression of TNF-α, IL-18, IL-6, and IL-1β in the Sham, GFS, and GFS + H151 groups (n = 5 eyes in each group). **l** Relative mRNA expression of fibronectin, CTGF, COL1A1, α-SMA, and COL3A1 in the Sham, GFS, and GFS + H151 groups (n = 5 eyes in each group). Results were expressed as mean ± SD. Statistical analysis was performed using log-rank test (**b**) and one-way ANOVA followed by Bonferroni’s post-hoc test (**g**, **h**, **k**, **l**). #, Welch’s ANOVA with Dunnett's T3 post-hoc test. *P < 0.05, **P < 0.01, ***P < 0.001. STING, stimulator of interferon genes; H151, STING inhibitor; Sham, sham-operated; α-SMA, α-smooth muscle actin; F4/80, macrophage marker; DAPI, 4′,6-diamidino-2-phenylindole; p-p65, phospho-p65; NF-κB, nuclear factor kappa-B; TNF-α, tumor necrosis factor-α; IL, interleukin; CTGF, connective tissue growth factor; COL1A1, collagen type I alpha 1; COL3A1, collagen type III alpha 1

**Fig. 7:**
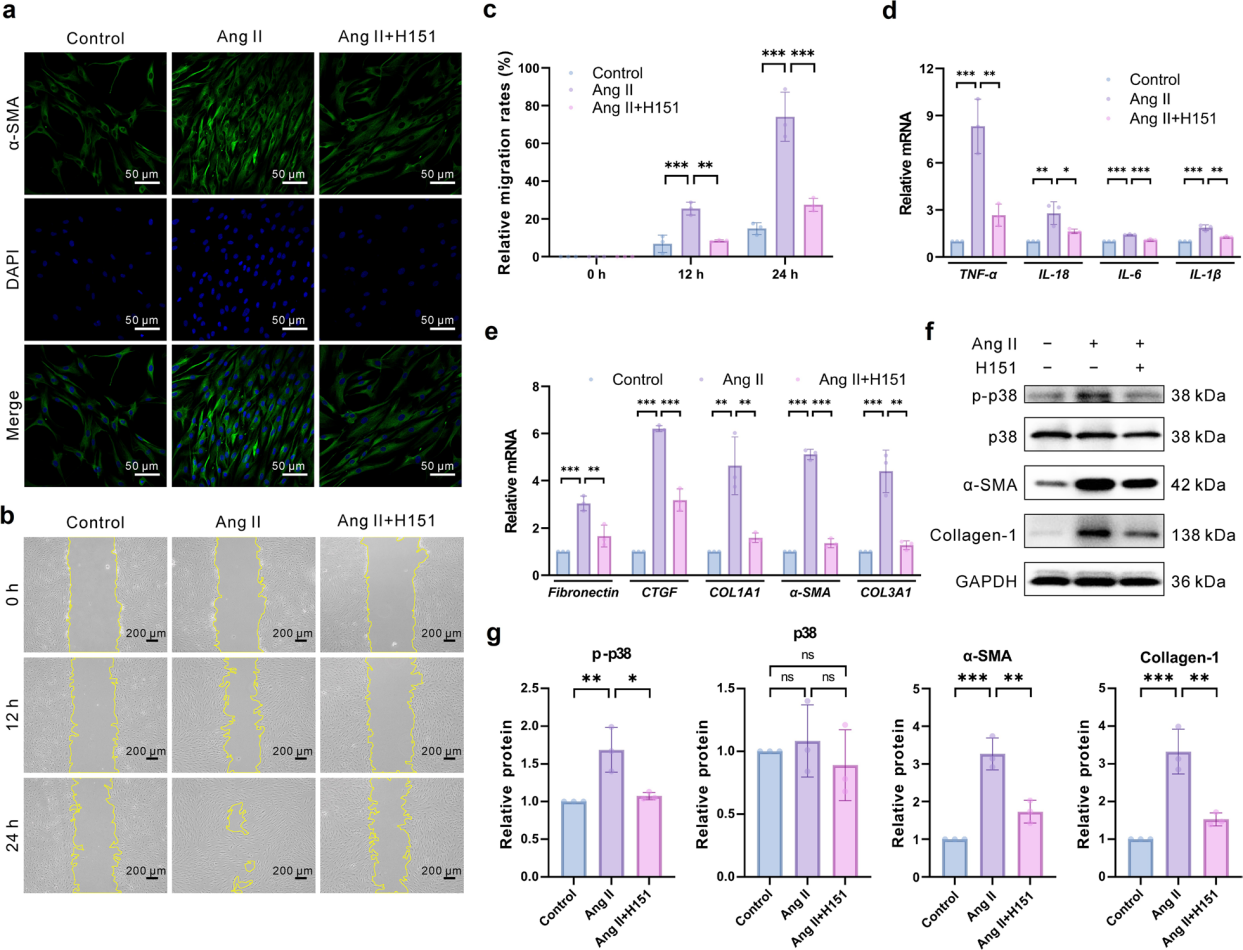
In vitro effects of H151 on inflammation and fibrosis of Ang II-induced HTFs. **a** Immunofluorescence staining of HTFs to identify α-SMA after the treatment with Ang II and Ang II + H151 (n = 3 eyes in each group). **b**, **c** Wound healing assay: cells were incubated with or without H151 2 h before Ang II (1 μM) treatment for 24 h. Subsequently, the wound scratches were imaged and quantified (n = 3 eyes in each group). **b** Representative images of the different treatment groups at different time points after the scratch. **c** Relative migration rates in each group. **d** Relative mRNA expression of TNF-α, IL-18, IL-6, and IL-1β in control, Ang II, and Ang II + H151 groups (n = 3 eyes in each group). **e** Relative mRNA expression of fibronectin, CTGF, COL1A1, α-SMA, and COL3A1 in control, Ang II, and Ang II + H151 groups (n = 3 eyes in each group). **f**, **g** Representative immunoblotting images: H151 significantly inhibited Ang II-induced p-p38, α-SMA, and collagen I protein expression by western blot (n = 3 eyes in each group). Results were expressed as mean ± SD. Statistical analysis was performed using one-way ANOVA followed by Bonferroni’s post-hoc test (**d**, **e**, **g**) and two-way ANOVA followed by Bonferroni’s post-hoc test (**c**). ns, not significant, *P < 0.05, **P < 0.01, ***P < 0.001. H151, STING inhibitor; Ang II, angiotensin II; HTFs, human Tenon’s capsule fibroblasts; α-SMA, α-smooth muscle actin; TNF-α, tumor necrosis factor-α; IL, interleukin; CTGF, connective tissue growth factor; COL1A1, collagen type I alpha 1; COL3A1, collagen type III alpha 1; p-p38, phospho-p38; DAPI, 4′,6-diamidino-2-phenylindole

Previous studies reported that the STING inhibitor H151 inhibits various inflammatory diseases, such as neovascular age-related macular degeneration, myocardial infarction, and psoriasis [19, 30, 31]. Based on this evidence, it was hypothesized that H151 may attenuate inflammation and fibrosis. Subconjunctival injection of H151 prolonged the survival of filtering blebs in WT mice (Fig. 6a, b). H&E staining (Fig. 6c) revealed that the thickness of the conjunctival Tenon’s capsule was reduced in GFS + H151 mice compared with GFS mice. Consistently, α-SMA fluorescence staining showed reduced fibrosis in GFS + H151 mice (Fig. 6d). Masson and Sirius red stainings further showed significantly increased collagen deposition in WT mice after GFS, and this fibrotic response was markedly reversed by a subconjunctival injection of H151 (Fig. 6e–h). Immunofluorescence staining for F4/80 and STING was performed to evaluate postoperative inflammation. Both markers increased after GFS, while H151 treatment effectively suppressed their postoperative induction (Fig. 6i). In addition, H151 significantly reduced p65 phosphorylation after GFS (Fig. 6j) and inhibited postoperative mRNA expression of inflammatory factors (TNF-α, IL-18, IL-6, and IL-1β) as well as fibrosis factors (fibronectin, CTGF, COL1A1, α-SMA, and COL3A1) (Fig. 6k, l). In vitro immunofluorescence staining showed that H151 reduced Ang II-induced α-SMA expression (Fig. 7a). Pre-treatment with H151 significantly inhibited Ang II-induced migration of HTFs (Fig. 7b, c). Moreover, H151 suppressed Ang II-induced mRNA expression of several inflammatory factors (TNF-α, IL-18, IL-6, and IL-1β) and fibrosis factors (fibronectin, CTGF, COL1A1, α-SMA, and COL3A1) (Fig. 7d–l). Finally, H151 inhibited Ang II-induced increase in p-p38, α-SMA, and collagen I protein expression compared with the Ang II group (Fig. 7m, n), which was consistent with the in vivo results seen in *Sting*^*−/−*^ mice.

## Discussion

STING, a critical protein in immunology and molecular biology, plays a central role in the body’s innate immune response to infections. STING helps the immune system in the detection and response to pathogens by triggering the production of interferons, which are signaling proteins that alert the immune system to the presence of harmful invaders. It is also closely associated with fibrosis in various systemic diseases. GFS is a commonly used treatment for several types of glaucoma. However, it frequently fails due to scar formation from excessive fibrosis of the filtration bleb after surgery. This study evaluated STING expression in clinical samples after GFS, and the results indicated its crucial role in scarring.

The relationship between inflammation and scar formation is complex and multifaceted. Inflammation not only precedes fibrosis but also predicts its severity [32, 33], and activation of the cGAS-STING pathway through type I IFNs and chemokines including IP-10, amplifies both processes [34, 35]. Conversely, excessive extracellular matrix (ECM) deposition stiffens the local microenvironment, promotes immune cell recruitment through integrin-mediated mechanotransduction, and reinforces a self-perpetuating inflammation-fibrosis cycle [36]. Together with our results that STING activated p38 pathway, these observations support an “inflammation-first, fibrosis-feedback” model underlying postoperative scarring after GFS. Immune cells are activated and recruited to the site of injury during inflammation, followed by the release of cytokines and chemokines that promote the activation of fibroblasts. Persistent activation of fibroblasts leads to excessive ECM deposition, which further disrupts tissue architecture, causing fibrosis, finally leading to dysregulated tissue repair [37, 38]. The inflammatory cytokines TNF-α and IL-6 stimulate the production of extracellular matrix components including collagen, further contributing to scar formation [39]. Moreover, the inhibition of inflammatory responses leads to a reduction in scar formation. For example, the use of anti-inflammatory drugs or the application of materials with anti-inflammatory properties during surgical procedures effectively decreases postoperative scarring [38]. These findings highlight the importance of inflammation in scar formation and suggest potential therapeutic strategies for scar prevention and treatment. Six significantly differentially expressed genes were found by integrating RNA-seq with qPCR validation: the inflammatory cytokines *IL-6, IL-1β, IL-18*, and *TNF-α*, together with the fibrogenic markers *COL1A1* and* COL3A1*. STING inhibition markedly down-regulated these inflammatory mediators, thereby attenuating the subsequent fibrotic response and limiting scar formation.

STING-related signaling pathways are associated with the occurrence of chronic inflammatory diseases in various organs and tissues. In liver fibrosis, cytosolic mitochondrial DNA (mtDNA) activates the cGAS–STING pathway, leading to IRF3 activation and the activation of the nucleotide-binding oligomerization domain-, leucine-rich repeat-, and pyrin domain-containing receptor 3 (NLRP3), and thus promote inflammation and fibrosis. X-box binding protein 1 (XBP1) promotes liver fibrosis progression by modulating mitochondrial autophagy and mtDNA leakage. Thus, the inhibition of STING signaling significantly alleviates liver fibrosis [40]. STING expression is elevated in the retina of patients with diabetic retinopathy (DR) and in diabetic mice. cGAS-STING activation is associated with retinal inflammation, endothelial cell senescence, and vascular lesions. This pathway promotes inflammation and cell senescence through the TBK1/IRF3 and nuclear factor kappa-B (NF-κB) pathways, playing a key role in DR [41]. A study using a retinal ischemia/reperfusion model revealed that the cGAS-STING pathway is activated in acute glaucoma, leading to inflammation and retinal ganglion cell death. Blocking the double-stranded DNA (dsDNA)-sensing cGAS with the STING inhibitor H151 suppresses the activation of the cGAS-STING pathway, thereby preventing microglial pyroptosis and significantly reducing retinal injury and retinal ganglion cell death after retinal ischemia–reperfusion [42]. This study found a high expression of STING in tissues after GFS, and the regulation of STING expression resulted in a modulation of ocular inflammation and scar formation. *Sting* gene knockout resulted in an attenuation of the filtering bleb scarring after GFS in a mouse GFS model. In addition, STING inhibition suppressed the MAPK pathway by interfering with p38 phosphorylation. This suggested that the p38 MAPK pathway regulated inflammation, in turn affecting scar formation. Beyond p38, STING activation rapidly engages the JAK-STAT1/3 axis through type I IFN [34, 43, 44] and regulates survival and metabolism through the PI3K-AKT pathway, with AKT providing feedback control over STING signaling amplitude [45, 46]. The contribution of these pathways to postoperative scar formation remains to be explored [47].

The relationship between p38 and glaucoma, as well as filtering bleb scarring after GFS, has been demonstrated in previous studies. p38 MAPK is a key node in the transforming growth factor-β (TGF-β) signaling pathway, which is involved in the deposition of ECM, inflammatory responses, and fibrosis. It is associated with inflammation triggered by glaucoma surgery. Its activation is associated with postoperative scar formation, thereby affecting the success rate of GFS [48]. The phosphorylation of p38 MAPK is significantly increased following GFS [48], and its inhibition improves the outcomes of GFS [49] including increased bleb survival, reduced scar formation, and better IOP control [49]. In addition, the phosphorylation of p38 plays a significant role in various ocular diseases, such as DR [50], glaucoma [51, 52], and dry eye [53]. Inhibition of p38 phosphorylation relieves the disease, to some extent, and improves the recovery.

Considering the clinical translation potential, the effect of the selective STING inhibitor H151 targeting both human and murine STING was assessed [19, 30, 31]. Postoperative scarring and p38 phosphorylation were alleviated in vitro and in vivo after the use of H151, suggesting a role in the inhibition of scar formation. Similar results were also observed in mice, in which the experimental group showed longer survival of filtration blebs than the control group. The alleviation of postoperative scarring and modulation of p38 phosphorylation suggested that H151 influenced inflammation and fibrosis involved in scar formation. The lower IOP and prolonged filtration bleb survival in the mouse experimental group further supported the clinical relevance of STING inhibition in glaucoma surgery. In this study, a clear therapeutic benefit was achieved after a single intraoperative administration of H151 during GFS. Clinical translation was supported by long-term dosing studies in animal models to define sustained efficacy and to characterize the safety profile of chronic STING inhibition. In parallel, comprehensive in vitro and in vivo investigations should be undertaken to delineate potential off-target and systemic effects and to facilitate the development of next-generation inhibitors with improved selectivity and targeted delivery, thereby minimizing adverse reactions and maximizing therapeutic benefit.

Our results demonstrated that STING is a central driver of postoperative inflammation and scarring after GFS, and that its suppression—genetic or pharmacologic—markedly reduces scar formation. These data position STING as a potential biomarker for predicting fibrosis risk and as a compass for personalizing anti-scarring therapy. Currently, translating STING-related findings into clinical practice faces multiple challenges, including the limitations of mouse models in reflecting human complexity and the need for multi-species or humanized models. Additionally, we recognize that the immune system is the first line of defense, and our KEGG data analysis revealed that inhibiting STING affects multiple immune pathways, including Th17-related differentiation. Although subconjunctival injection results in minor absorption, using STING-related therapies may present challenges for patients with immune abnormalities.

## Conclusion

STING is markedly up-regulated in Tenon’s capsule fibroblasts after GFS, triggered by excess Ang II and culminating in p38 MAPK phosphorylation. Genetic or pharmacologic (H151) blockade of this axis suppresses inflammatory and fibrogenic mediators, prolongs bleb survival and reduces collagen deposition without evident toxicity. Our findings collectively identify STING as a key therapeutic target for preventing filtering bleb scarring and position its selective inhibitor H151 as a promising drug candidate for anti-fibrotic therapy in GFS.

## Supplementary Information

**Additional file 1.**

## Data Availability

The RNA-seq data generated in this study have been deposited in the NCBI Sequence Read Archive (SRA) under BioProject accession PRJNA1368288. All other data supporting the findings of this study are available from the corresponding author upon reasonable request.
