# Supplementary material for: STING deficiency alleviates scar formation after glaucoma filtration surgery by suppressing p38 MAPK-induced inflammation in mice
Source: Eye Vis (Lond). 2026 Feb 2;13:7. doi: 10.1186/s40662-026-00475-3 (PMC12862908; doi:10.1186/s40662-026-00475-3)
Supplement: Supplementary file 1 — Additional file 1. [file 40662_2026_475_MOESM1_ESM.docx]

**Supplementary Materials**

**Supplementary Tables**

**Table S1. Quantitative polymerase chain reaction (qPCR) primers sequences.**

| **Gene** | **Forward primer** | **Reverse primer** |
| --- | --- | --- |
| human *Sting* | GGTGGCTTGAGGGGAAC | TGGAGTGGGGCATCTTCT |
| human *IL-6* | ACTCACCTCTTCAGAACGAATTG | CCATCTTTGGAAGGTTCAGGTTG |
| human *IL-1β* | TCATCTTTCAACACGCAGGACAGG | GGACAGGATATGGAGCAACAAGTGG |
| human *IL-18* | TGGAATCAGATTACTTTGGCAAGC | CCTTGGTCAATGAAGAGAACTTGG |
| human *TNF-α* | CCCAGGGACCTCTCTCTAATC | ATGGGCTACAGGCTTGTCACT |
| human *COL1A1* | TGCTGGCAAAGATGGAGAGG | GGGCCTTGTTCACCTCTCTC |
| human *COL3A1* | CTCAGGGTGTCAAGGGTGAAAGTG | TGTACCAGCCAGACCAGGAAGAC |
| human *α-SMA* | CTACTGCTGAGCGTGAGATTGTC | CCATCAGGCAACTCGTAACTCTTC |
| human *Fibronectin* | GGCGACAGGACGGACATCTTTG | GGCACAAGGCACCATTGGAATTTC |
| human *CTGF* | CGGGAAATGCTGCGAGGAGTG | GGTCTGGGCCAAACGTGTCTTC |
| human *GAPDH* | AACGGATTTGGTCGTATTGG | TTGATTTTGGAGGGATCTCG |
| mouse *Sting* | CCAAGAACCCACAGACGGAAACAG | GGAGGAGGTGCCACTGAGGTC |
| mouse *IL-6* | TGGGACTGATGCTGGTGACA | ACAGGTCTGTTGGGAGTGGT |
| mouse *IL-1β* | TGAAGGGCTGCTTCCAAACC | GCCTGCCTGAAGCTCTTGTT |
| mouse *IL-18* | GTGAACCCCAGACCAGACTG | CCTGGAACACGTTTCTGAAAGA |
| mouse *TNF-α* | GACTACGTGCTCCTCACCCA | TCTTGACGGCAGAGAGGAGG |
| mouse *COL1A1* | TGGTCCTGCTGGTCCTGCTG | GTCACCTTGTTCGCCTGTCTCAC |
| mouse *COL3A1* | ACCACCAGGTCCTAGAGGAAACAG | CAGCAGCACCACCACCACAG |
| mouse *α-SMA* | CAGGGAGTAATGGTTGGAATGGG | AGTTGGTGATGATGCCGTGTTC |
| mouse *Fibronectin* | AGTGGCTGAAGTCGCAAGGAAAC | TAAGTCTGGGTCACGGCTGTCTC |
| mouse *CTGF* | CACCGCACAGAACCACCACTC | AATGGCAGGCACAGGTCTTGATG |
| mouse *GAPDH* | TCACCATCTTCCAGGAGCGAGAC | AGACACCAGTAGACTCCACGACATAC |

**Table S2. Antibodies and dilutions used in this study.**

| Antibody | Dilutions for WB* | Dilutions for IF or IHC* | Company | Catalogue No. |
| --- | --- | --- | --- | --- |
| Rabbit anti p-p38 | 1:1000 |  | ProteinTech | 28796-1-AP |
| Rabbit anti p38 | 1:1000 |  | ProteinTech | 14064-1-AP |
| Rabbit anti α-SMA | 1:1000 | 1:200 | ProteinTech | 14395-1-AP |
| Rabbit anti collagen-Ⅰ | 1:1000 |  | ProteinTech | 14695-1-AP |
| Rabbit anti STING | 1:1000 | 1:200 | Cell Signaling Technology | 13647 |
| Rabbit anti p-TBK1 |  | 1:200 | Cell Signaling Technology | 5483 |
| Rabbit anti p-IRF3 |  | 1:200 | Cell Signaling Technology | 29047 |
| Rabbit anti p-p65 |  | 1:200 | Cell Signaling Technology | 3033 |
| Rabbit anti F4/80 |  | 1:200 | Epizyme | R013726 |
| Rabbit anti GAPDH | 1:5000 |  | ProteinTech | 10494-1-AP |
| Goat Anti-Rabbit IgG HRP | 1:5000 | 1:500 | Proteintech | SA00001-2 |
| Goat Anti-Mouse IgG HRP | 1:5000 | 1:500 | Proteintech | SA00001-1 |
| Alexa Fluor 594 Goat anti-Rabbit IgG |  | 1:500 | Abcam | ab150080 |
| Alexa Fluor 488 Goat Anti-Rabbit IgG |  | 1:500 | Abcam | ab150077 |
| Alexa Fluor 488 Goat Anti-Mouse IgG |  | 1:500 | Abcam | ab150113 |

WB = Western blotting; IF = immunofluorescence; IHC = immunohistochemistry; p-p38 = phospho-p38; α-SMA = α-smooth muscle actin; STING = stimulator of interferon genes; p-TBK1 = phospho-tank-binding kinase 1; p-IRF3 = phospho-interferon regulatory factor 3; p-p65 = phospho-p65; F4/80 = macrophage marker; GAPDH = glyceraldehyde 3-phosphate dehydrogenase; HRP = horseradish peroxidase

*: The diluent of for the antibodies was Universal Antibody Dilution Buffer (PS119, Epizyme, China).

**Table S3. Indiana bleb appearance grading scale of filtering bleb after glaucoma filtration surgery (GFS) in wild-type (WT)+GFS and Sting***^−/−^***+GFS.**

| **Group** | **Day 1** | **Day 7** | **Day 14** | **Day 28** |
| --- | --- | --- | --- | --- |
| WT+GFS | H3, E3, V0 | H2, E1, V2 | H0, E0, V2 | H0, E0, V3 |
| Sting*^−/−^*+GFS | H3, E3, V0 | H3, E2, V1 | H2, E2, V1 | H2, E2, V1 |

H = height, H0 (flat) to H4 (high); E = extent, E0 (less than 1 clock hour) to E3 (more than 4 clock hours); V = vascularity, V0 (avascular) to V4 (extensive vascularity). n = 5 eyes in each group.

**Table S4. Indiana bleb appearance grading scale of filtering bleb after glaucoma filtration surgery (GFS) in wild-type (WT)+GFS and Sting***^−/−^***+GFS.**

| **Group** | **Day 1** | **Day 7** | **Day 14** | **Day 28** |
| --- | --- | --- | --- | --- |
| WT+GFS | H3, E3, V0 | H2, E1, V2 | H1, E1, V2 | H0, E0, V3 |
| H151+GFS | H3, E3, V0 | H2, E2, V1 | H2, E2, V1 | H2, E2, V1 |

H = height, H0 (flat) to H4 (high); E = extent, E0 (less than 1 clock hour) to E3 (more than 4 clock hours); V = vascularity, V0 (avascular) to V4 (extensive vascularity). n = 5 eyes in each group.

**Table S5. Raw data of intraocular pressure.**

| Group  Wild-type: 1  *Sting**^−/−^*: 0 | Intraocular pressure (mmHg) | | | |
| --- | --- | --- | --- | --- |
|  | Baseline | Immediately after surgery | 7 days after surgery | 28 days after surgery |
| 1 | 9 | 6 | 9 | 10 |
| 1 | 10 | 6 | 8 | 11 |
| 1 | 9 | 7 | 10 | 10 |
| 1 | 11 | 6 | 9 | 11 |
| 1 | 12 | 8 | 8 | 10 |
| 1 | 11 | 8 | 9 | 10 |
| 1 | 11 | 7 | 11 | 11 |
| 1 | 13 | 7 | 10 | 11 |
| 1 | 12 | 5 | 8 | 6 |
| 1 | 10 | 6 | 9 | 6 |
| 0 | 12 | 6 | 9 | 10 |
| 0 | 11 | 7 | 8 | 11 |
| 0 | 12 | 6 | 10 | 10 |
| 0 | 13 | 7 | 10 | 11 |
| 0 | 10 | 6 | 12 | 12 |
| 0 | 13 | 6 | 11 | 10 |
| 0 | 13 | 6 | 13 | 11 |
| 0 | 8 | 6 | 9 | 11 |
| 0 | 11 | 7 | 9 | 10 |
| 0 | 13 | 5 | 13 | 10 |

**Supplementary Figures**

**Figure S1.** PCA and correlation analyses reveal differential molecular profiles in the GFS mouse model. **a** PCA Score Plot Distribution of samples from the Sham, WT+GFS and *Sting^−/−^*+GFS groups along PC1 and PC2 in PCA space. **b** Correlation Heatmap Pairwise Pearson correlation coefficients among the Sham, WT+GFS and *Sting^−/−^*+GFS groups. The color scale (blue to red) represents correlation values from 0.80 to 1.00. PCA, principal component analysis; GFS, glaucoma filtration surgery; WT, wild-type; Sham, sham-operated

**
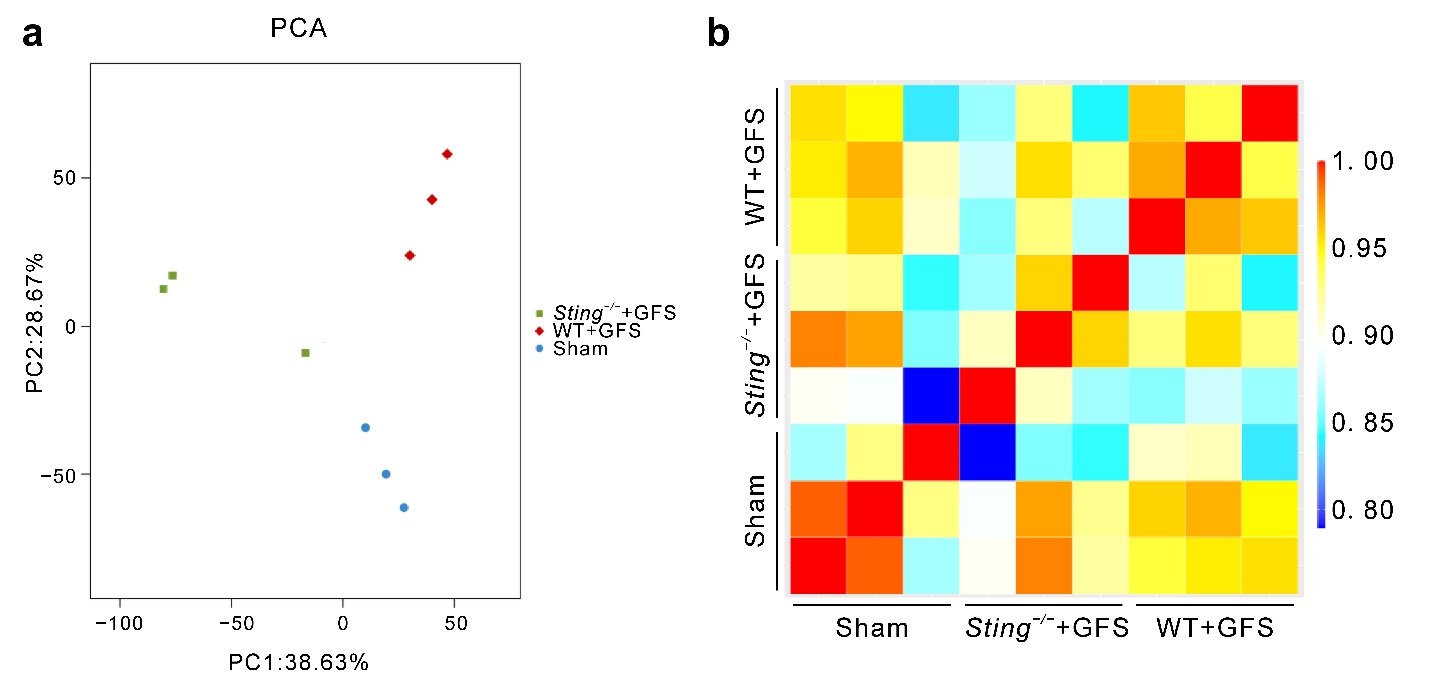
**

**Figure S2.** In vitro effects of SB203580 on inflammation and fibrosis of Ang Ⅱ-induced HTFs. **a**–**b** Wound healing assay: cells were incubated with or without SB203580 (10 µM) 2 h before Ang Ⅱ (1 µM) treatment for 24 h. Subsequently, the wound scratches were imaged and quantified (n = 3 eyes per group). **a** Representative images of the different treatment groups at different time points after the scratch. **b** Relative migration rates in each group. **c**–**d** Concentrations of IL-6 and TNF-α in the control, Ang Ⅱ, and Ang Ⅱ+SB203580 groups detected by ELISA (n = 3 eyes per group). **e**–**f** Representative immunoblotting images: SB203580 significantly inhibited Ang Ⅱ-induced α-SMA and collagen-Ⅰ protein expression by Western blot (n = 3 eyes per group). Results were expressed as mean ± SD. Statistical analysis was performed using one-way ANOVA followed by Bonferroni’s post-hoc test in (**b, c, d, f**). ***P* < 0.01, ****P* < 0.001. Ang Ⅱ, angiotensinⅡ; HTF, human Tenon’s capsule fibroblasts; IL, interleukin; TNF-α, tumor necrosis factor-α; α-SMA, α-smooth muscle actin; GAPDH, glyceraldehyde 3-phosphate dehydrogenase

**
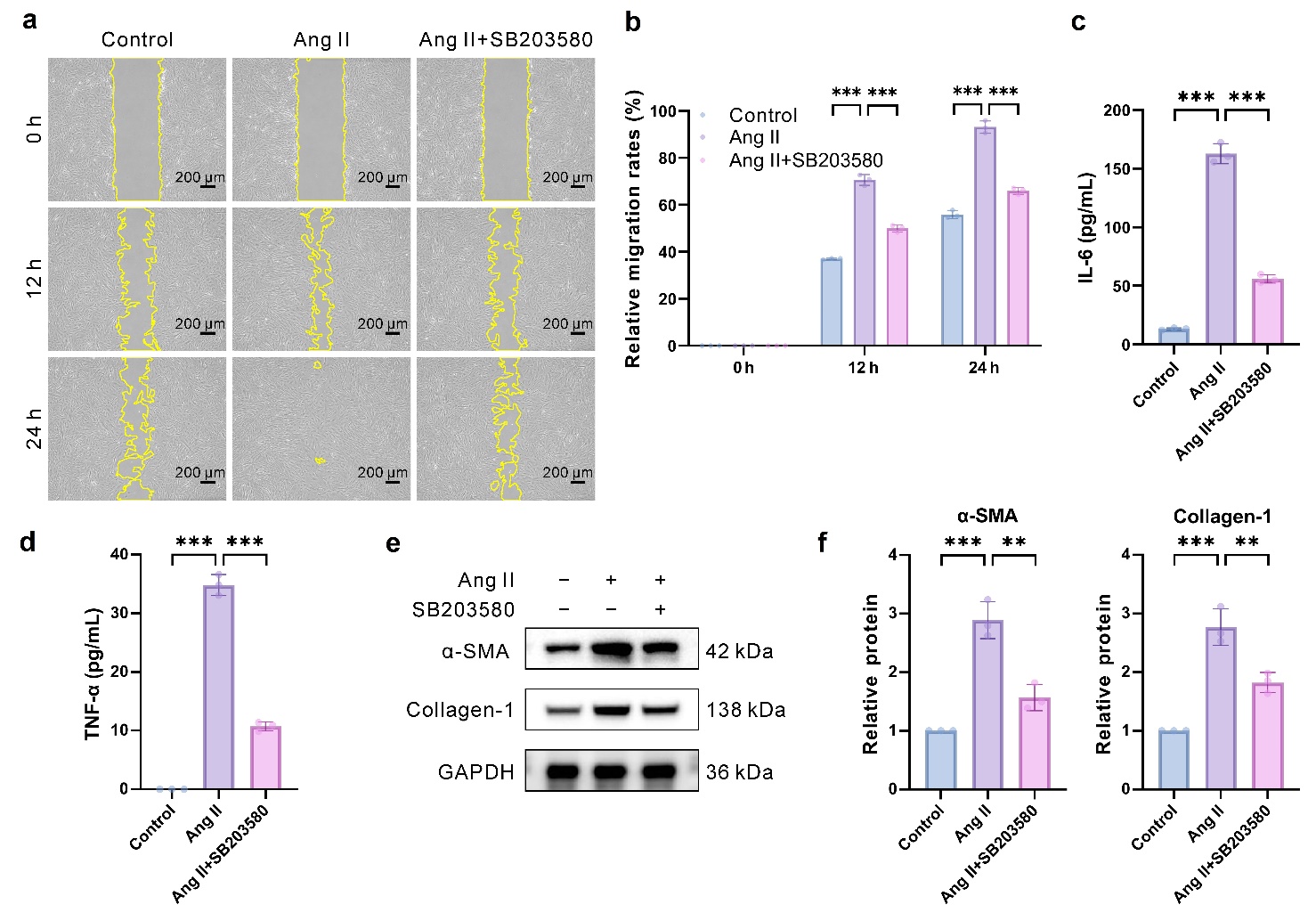
**

**Supplementary Methods**

**Animal randomization and masking procedures**

Mice were divided into groups by simple randomization using a random-number table. Each mouse received an ear-mark identifier and the grouping information was saved as an encrypted file by a researcher who did not participate in subsequent procedures. Cage cards displayed only color-coded labels (no text), and the grouping information was available to all researchers only after data analysis was complete. Whenever feasible, a double-blind design was maintained: the same investigator prepared and administered drugs (still blinded to group by color code), whereas histological evaluation, image acquisition and data analyses were performed independently by two other investigators who remained blinded to the grouping information. All image and data files were labelled with numeric codes and decoding was only carried out after data analyses were completed.

**Inclusion and exclusion criteria of animals and human subjects**

For animals:

Inclusion: C57BL/6J and *Sting^−/−^* male mice, 7–8 weeks old.

Exclusion: any mouse with congenital or acquired ocular abnormalities or ocular infection (e.g., eyelid defect, corneal leukoma, infectious keratitis).

For human samples:

Inclusion: patients aged 30–60 years old and scheduled for strabismus surgery, enucleation surgeries, or secondary glaucoma surgery owing to scarring following previous glaucoma filtration surgery.

Exclusion: previous ocular surgery, uveitis, ocular trauma, or chronic use of tropic drops (> 4 weeks).

**The frequency of mycoplasma testing for cells**

Cells were tested for mycoplasma contamination immediately after the human Tenon’s capsule fibroblasts were successfully cultured and then once every month. Additional tests were performed at any time whenever morphological abnormalities or unexpected growth kinetics were observed.
